# The Effect of Selective Laser Melting Process Parameters on the Microstructure and Mechanical Properties of Al6061 and AlSi10Mg Alloys

**DOI:** 10.3390/ma12010012

**Published:** 2018-12-20

**Authors:** Ahmed H. Maamoun, Yi F. Xue, Mohamed A. Elbestawi, Stephen C. Veldhuis

**Affiliations:** Department of Mechanical Engineering, McMaster University, 1280 Main Street West, Hamilton, ON L8S 4L7, Canada; xueyf4@mcmaster.ca (Y.F.X.); veldhu@mcmaster.ca (S.C.V.)

**Keywords:** additive manufacturing, selective laser melting, AlSi10Mg, Al6061, SLM process parameters, quality of the as-built parts

## Abstract

Additive manufacturing (AM) offers customization of the microstructures and mechanical properties of fabricated components according to the material selected and process parameters applied. Selective laser melting (SLM) is a commonly-used technique for processing high strength aluminum alloys. The selection of SLM process parameters could control the microstructure of parts and their mechanical properties. However, the process parameters limit and defects obtained inside the as-built parts present obstacles to customized part production. This study investigates the influence of SLM process parameters on the quality of as-built Al6061 and AlSi10Mg parts according to the mutual connection between the microstructure characteristics and mechanical properties. The microstructure of both materials was characterized for different parts processed over a wide range of SLM process parameters. The optimized SLM parameters were investigated to eliminate internal microstructure defects. The behavior of the mechanical properties of parts was presented through regression models generated from the design of experiment (DOE) analysis for the results of hardness, ultimate tensile strength, and yield strength. A comparison between the results obtained and those reported in the literature is presented to illustrate the influence of process parameters, build environment, and powder characteristics on the quality of parts produced. The results obtained from this study could help to customize the part’s quality by satisfying their design requirements in addition to reducing as-built defects which, in turn, would reduce the amount of the post-processing needed.

## 1. Introduction

Additive manufacturing (AM) is considered one of the leading sectors of the upcoming industrial revolution “Industry 4.0” [1]. The AM of metals using selective laser melting (SLM) promises significant development of a variety of critical applications in different industrial fields [2]. The AM of Al alloys could produce high-performance lightweight components with relatively high material quality, mechanical properties, and design flexibility. The selection of the SLM process parameters plays an essential role in controlling the material and mechanical properties of products customized according to their function and design requirements. The effect of the SLM process parameters on the quality of Al alloys was previously presented in some studies [3,4,5,6,7,8,9]. Prashanth et al. [10] reported that the selected strategy of hatch spacing and contour parameters could significantly affect the microstructure and mechanical properties of the AlSi12 parts fabricated using SLM. Their results showed that applying the contour parameters negatively affects the ductility of parts due to residual stresses along the surface. However, the effect of the SLM parameters on the microstructure of the AlSi12 parts was not reported in that study. Biffi et al. [8] studied the influence of the SLM energy density on the mechanical properties of the AlSi10Mg samples. However, the effect of each process parameter was not studied to detect how the leading parameter affects the microstructure and mechanical properties. Li et al. [11] reported that the high cooling rate during the SLM process of AlSi10Mg results in ultrafine-grained microstructures, and thus leads to superior mechanical properties as compared to casted material of the same alloy. The microstructure characteristic could be affected by the applied cooling rate according to the selected SLM parameters. Akram et al. simulated a model of grain structure evolution in the multi-layer deposition during the SLM process [12]. Their results illustrated the change in grain size and orientation according to the process parameters applied. 

In SLM of Al alloys, the chemical composition of the Al alloys could result in variation between their microstructure and mechanical properties, due to the difference in some elements such as Si and Mg. However, SLM of some Al alloys, such as Al6061, results in solidification and liquation cracking due to the material’s relatively higher coefficient of thermal expansion (CTE) [3]. This is why AlSi10Mg is the most commonly-used Al alloy for the SLM process due to its lower CTE compared to the Al6061 alloy [13]. The Si content may also play a significant role in microstructure evolution and the elimination of hot cracks. Uddin et al. [14] reported that preheating the build plate at 500 °C using specific process parameters resulted in crack-free parts. However, the mechanical properties of the fabricated components are adversely affected as compared to as-built parts produced without preheating the build platform. Martin et al. [15] studied the effect of adding nanoparticles of some additives to Al6061 and Al7075 alloy powder. Their results showed that the additives added could control the solidification process and reduce the crack formation inside the as-built parts. However, lower mechanical properties were obtained as compared to the as-built components of the original alloy due to the formation of spherical pores. Moreover, the effect of the SLM process parameters for Al6061 alloy was not reported in the literature to investigate the ability to control the hot-crack formation. 

Fulcher et al. [13] reported that the SLM process map should be regularly updated for each material as technical capabilities develop. This could help to optimize the SLM process parameters and customize the characteristics of the as-built parts. Consequently, the microstructure and mechanical properties of the additively-manufactured parts can be tailored according to their design requirements. In general, the literature studies show the importance to update the SLM process map according to the capabilities of the upgraded machines. This might help to detect the leading parameter affecting each characteristic to achieve the desired quality of the fabricated parts. However, the laser power of SLM was limited to 200 W, which is a relatively low figure compared to currently-used laser power which can reach 400 W for the majority of existing machines. Therefore, due to the widespread use of Al6061 in the aerospace and automotive fields, study is recommended of the influence of SLM process parameters on this material. This also might reduce the amount of post-processing applied to heal the as-built part defects. 

The current study focuses on the effect of SLM process parameters on microstructure and mechanical properties of both AlSi10Mg and Al6061 as-built parts. This work completes the comprehensive study presented by Maamoun et al. [16] to develop a full process map for different Al alloys fabricated with SLM. Microstructure characterization is performed to investigate the evolution of as-built microstructure along with changing the SLM process parameters. An optimum process parameter range is investigated to reduce the microstructure defects and hot-cracks formation. The behavior of mechanical properties is studied for both materials. A regression model is created based on the design of experiment (DOE) analysis for each mechanical property along the applied range of SLM process parameters. The regression model trend for each property of the as-built parts is validated according to experimental results, and is additionally verified with microstructure analysis. A comparison between the obtained results from the current study as compared to literature is conducted to illustrate the effect of powder characteristics, build environment, and process parameters on the properties of parts fabricated. This study presents a process map of the influence of SLM process parameters of AlSi10Mg and Al6061 as-built parts, and this could offer the following:

1. Customization of parts fabricated according to their function and design requirements.
2. Reduction or elimination of the microstructure defects by investigating the optimum range of process parameters, which could reduce the amount of post-processing required.

## 2. Experimental Procedure

In the current study, the samples were produced using the SLM process parameters listed in Table 1 and Table 2. The methodology of the DOE is the same as reported by Maamoun et al. [16]. The technique of one factor at a time (OFAT) is applied for AlSi10Mg parts, and the response surface method is used for Al6061 parts. The correlation coefficient (R^2^) is used to indicate how the regression models fit with the measured data; this factor was added to each mechanical property characteristic map for each material. The build plate was preheated to 200 °C before building started under an argon medium. So, AlSi10Mg_200C and Al6061_200C also referred to the as-built AlSi10Mg and Al6061 samples respectively. Powder characteristics, microstructure analysis and the measurement of mechanical properties were performed with the following methods. 

### 2.1. Powder Characteristics

ASTM F3049-14 was used to examine the fresh powder of AlSi10Mg and Al6061 after sieving using a 75 µm mesh. The full powder characterization of the same powders used in this study was reported by Maamoun et al. [16]. A particle size distribution test showed a particle size ranges from 12 to 120 µm for Al6061 and from 12 µm to 110 µm for AlSi10Mg. Powder morphology of a spherical particle shape was detected with the existence of some elongated particles that might affect the flowability. Both powders have a positively skewed profile which could result in a higher density and better surface roughness as compared to Gaussian and negatively-skewed powder distribution [17]. 

### 2.2. Microstructure Characterization

The microstructure of both AlSi10Mg and Al6061 as-built samples were characterized with optical microscopy (OM), scanning electron microscope (SEM), and X-ray diffraction (XRD) measurements. A Nikon optical microscope LV100 was used to evaluate the microstructure of the etched parts. The polishing and etching procedures were performed according to the recommendations of Maamoun et al. [18]. A TESCAN VP SEM equipped with an energy dispersive X-ray spectroscopy (EDS) detector, was used to investigate the grain size and structure observations. A Bruker D8 DISCOVER XRD instrument provided with a cobalt sealed tube source was used for the samples’ phase analysis. The XRD phase pattern was obtained for each sample along different orientations of the AlSi10Mg and Al6061 samples. 

### 2.3. Mechanical Properties Measurements

The microhardness measurement was performed according to ASTM E384-17 using an automatic Clemex CMT tester. The average values of the samples’ microhardness were obtained along the building direction (Z-direction) and along the plane parallel to the deposited layers (XY-plane). Each recorded value was an average of 5–10 indentations along the tested area of a 200 gf load applied over a 10 s dwell time. The residual stress was measured by an XRD instrument using a Vantec500 area detector, and the results were analyzed using LEPTOS software. The tensile rod samples were designed and fabricated according to the geometry and dimension included in ASTM E8/E8M-16a. The tensile test was performed according to ASTM E8 standard procedures using an MTS Criterion 43 universal test system which applies a load capacity up to 50 kN. 

## 3. Results and Discussion

### 3.1. Microstructure

**Figure 1:**
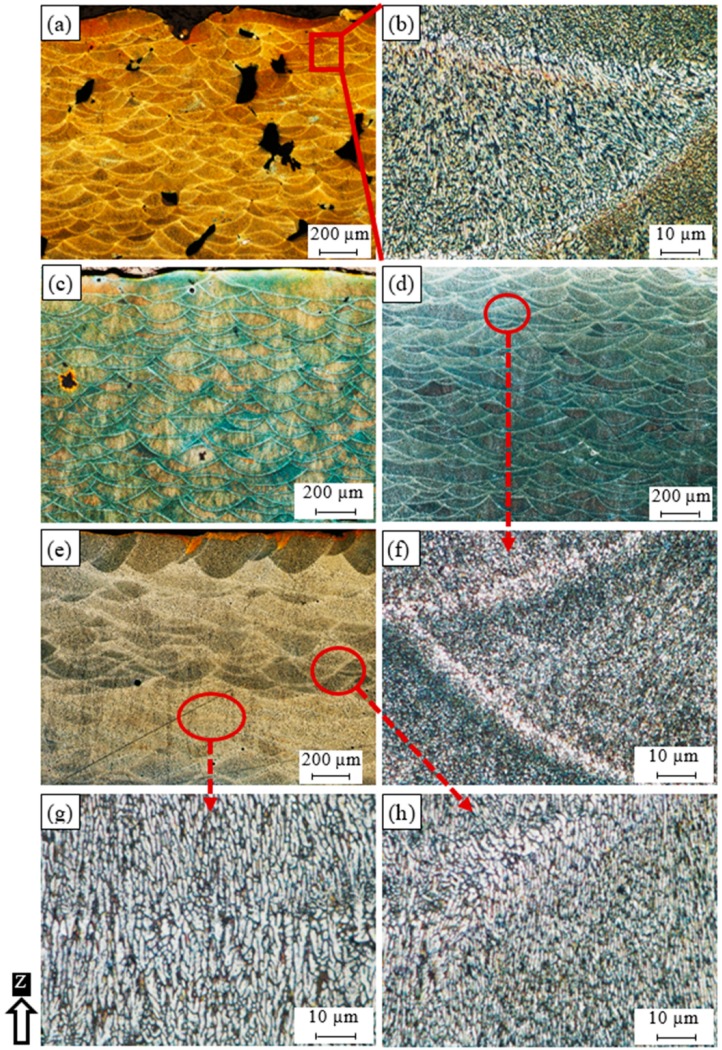
Microstructure of the as-built AlSi10Mg_200C samples processed under different SLM process parameters; (**a**,**c**) AS8; (**b**) AS7; (**d**,**f**) AS3, and (**e**,**g**,**h**) AS1.

The optical microscope analysis was performed using the as-built etched samples of AlSi10Mg and Al6061. Figure 1 shows the microstructure defects and observations along the building direction (Z-direction) of AlSi10Mg samples fabricated at different SLM process parameters. Figure 1a illustrates that process-induced porosity or keyhole pores of 100–250 µm size and irregular shapes are formed inside the AS8 sample fabricated at a low energy density of 27 J/mm^3^. This results from a lack of fusion due to insufficient powder delivery to the melted layer. Unmelted powder may be visible around these keyhole pores [19]. Figure 1a also shows that the melt pool solidified with an elliptically shaped profile, and that these melt pool shapes overlap in a specific arrangement according to the value of hatch spacing used. This shape is related to the Gaussian distribution of laser beam power [18]. Figure 1b shows a magnified view of the melt pool shape; a fine grain structure is observed inside, while a coarse grain is formed along its borders due to the gradient change of the solidification rate. Figure 1c shows the microstructure of the AS7 sample fabricated at an energy density of 38 J/mm^3^. The keyhole pore density and size are decreased due to a higher energy density. The melt pool shape geometry of the AS7 sample is enlarged compared to the AS8 sample due to a diminishing solidification rate together with an energy density increase. In the AS3 sample produced at a 50 J/mm^3^ energy density, the keyhole pores almost disappeared as shown in Figure 1d. A coarser grain structure is also present inside and along the borders of the melt pool shape as illustrated in Figure 1f. At a higher rate of energy density of 65 J/mm^3^ applied to the AS1 sample, the melt pool borders disappear along some layers, and spherical hydrogen pores can be seen in Figure 1e. The areas where the melt pool borders disappear show a more homogeneous structure with elongated columnar grains oriented along the building direction, Figure 1g. While the areas displaying melt pool borders show the same inhomogeneity of microstructure as in the other samples, they have a larger grain structure, as illustrated in Figure 1h. It is worthwhile to note that the energy density level significantly affects the solidification rate, and thus, creates specific microstructure characteristics corresponding to the applied values [20]. Also, according to the SLM process parameters listed in Table 1 for each sample, the low laser power of 200 W applied to the AS8 sample resulted in low energy density, and thus a lack of fusion according to the definition of the energy density in the following equation:

(1) $$E_{d}=\frac{P}{V_{s}{*D}_{h}{*T}_{l}}$$

where E_d_ represents energy density in J/mm^3^, P is the laser beam power (W), V_s_ is the laser scan speed (mm/s), D_h_ is the hatch spacing between scan passes, T*_l_* is the deposited layer thickness, which remains a constant value in this study with a 30 µm height. The disappearance of the melt pool profile borders observed inside the AS1 sample might be related to the reduction of the scan speed and hatch spacing parameters.

**Figure 2:**
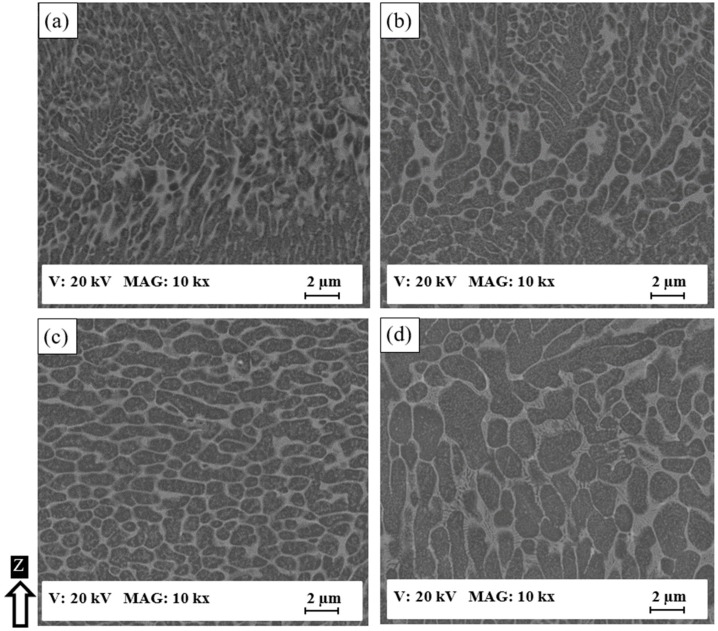
The SEM observations of the as-built AlSi10Mg microstructure along Z-direction; (**a**) AS8; (**b**) AS3; (**c**) AS1 near top surface, and (**d**) AS1 near the center.

**Figure 3:**
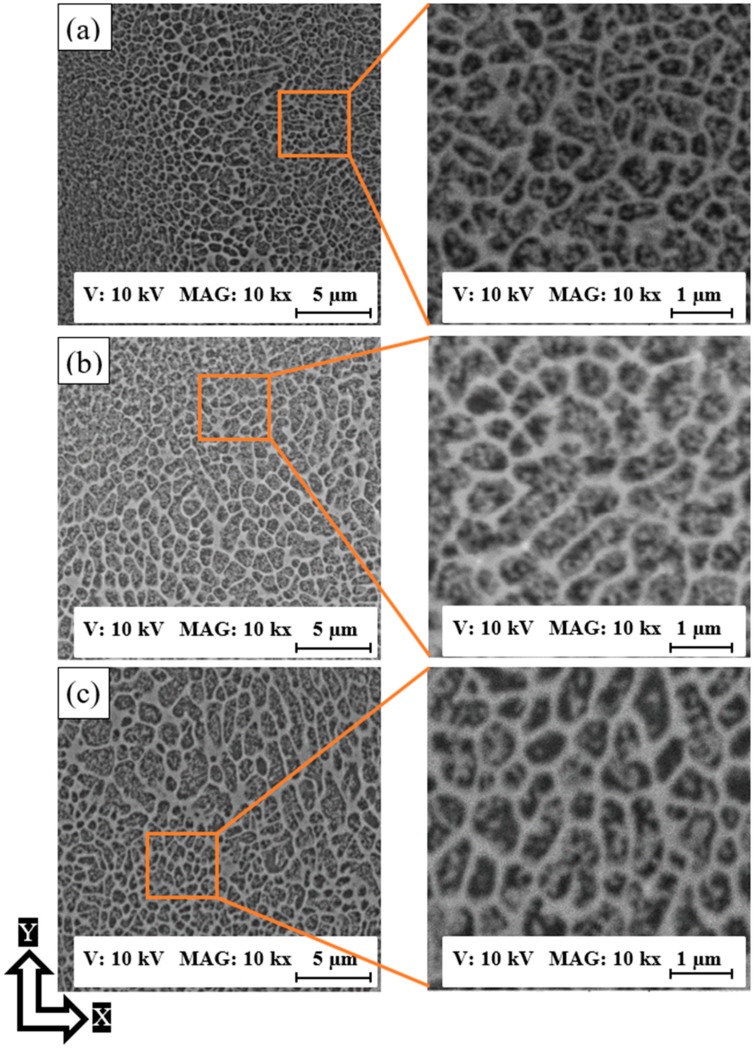
The SEM observations of the as-built AlSi10Mg microstructure along the XY plane; (**a**) AS8; (**b**) AS3, and (**c**) AS1.

SEM observations in Figure 2 and Figure 3 show the change in the developed microstructure and the evolution of the Al matrix grain size of the as-built AlSi10Mg samples produced at different energy densities and SLM process parameters. Figure 2 displays the microstructure along the Z-direction of the AlSi10Mg samples. In general, the development mechanism of the as-built AlSi10Mg microstructure depends on the mechanism of particle accumulated structure (PAS) formation [21]. The PAS mechanism shows that during the high cooling rate of 106–108 °C/s, Si is ejected out of the solidifying Al matrix to form a fibrous Si network around the Al matrix grain borders. At a lower energy density of 27 J/mm^3^, the microstructure shows an ultra-fine elongated grain structure with an inhomogeneous size distribution of Al matrix grains surrounded by a fibrous Si network. The Al matrix grain size ranges from 0.2 µm to 2 µm, where the finer grain structure formed inside the melt pool shape and larger grains located around its borders as displayed in Figure 2a. The increase of energy density to 50 J/mm^3^ results in the same microstructure formation with a coarser inhomogeneous microstructure and grain size ranging from 500 nm to 3 µm as illustrated in Figure 2b. Figure 2c,d shows that when the AS1 sample is produced at a higher energy density of 65 J/mm^3^, an equiaxed larger grain structure is present with Al matrix grain size varying between 3–4 µm. A more homogeneous microstructure is also obtained compared to the samples produced at a lower energy density. The final top layers in Figure 2c have a finer microstructure compared to the vicinity of the middle of the part in Figure 2d. This is attributed to the thermal gradient difference between these areas during the building of the layers, which affects the solidification rate.

The as-built AlSi10Mg samples along the XY plane had an equiaxed grain microstructure, as can be seen in Figure 3. The microstructure is inhomogeneous due to the existence of coarser grains along the border of the melt pool profile compared to the microstructure inside. This confirms the PAS formation mechanism of the microstructure development along the XY plane as well as the Z-direction [3,21]. Figure 3a shows the microstructure of the AS8 sample, where an inhomogeneous grain distribution of 0.15–1 µm size can be seen within the fine and coarser Al matrix grain zone. The grain size slightly increased along with energy density. Figure 3b presents the microstructure of the AS3 sample with a grain size ranging from 0.3 µm to 2 µm. The microstructure evolution of the higher energy density of 65 J/mm^3^ applied to the AS1 samples has almost the same Al matrix grain structure value, as illustrated in Figure 3c. The application of energy densities higher than 50 J/mm^3^ caused no significant difference in the microstructure. However, the XRD measurements were performed for a more accurate analysis of crystal size change and solubility percentage of the Si inside the Al matrix [22,23]. The zoom-in views of Figure 3a–c show more details about the evolution of the Al matrix grain inside the melt pool profile, which increases along with the energy density applied. The observed evolution of the Al Matrix grain size might be related to the reduction of solidification rate along with increasing the energy density [24].

**Figure 4:**
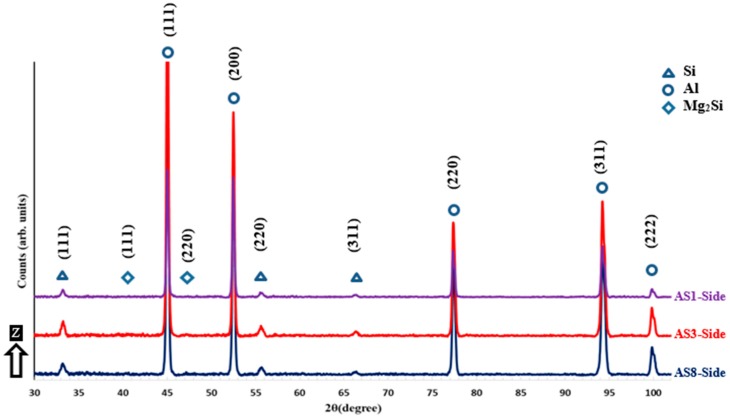
XRD phase pattern measured on the side surface (along the Z-direction) of different as-built AlSi10Mg samples.

**Figure 5:**
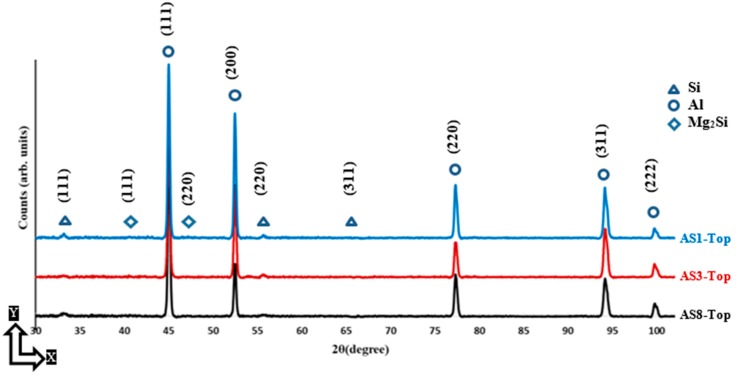
XRD phase pattern measured on the top surface (along the XY plane) of different as-built AlSi10Mg samples.

The XRD phase pattern presented in Figure 4 and Figure 5 shows a comparison of the Al and Si peak characteristics of different AlSi10Mg samples. The Al and Si peak is identified using the Joint Committee on Powder Diffraction Standards (JCPDS) patterns of 01-089-2837, 01-089-5012, respectively. A small peak of Mg_2_Si is detected according to the JCPDS pattern of 00-001-1192, and the low intensity of this peak is related to the existence of nano-size Mg_2_Si precipitates of 20–40 nm that are hardly detectable with XRD [18,23]. The difference in Al and Si peak width between the samples indicates crystal size change under different SLM process parameters. This can be inferred from Scherrer’s equation, where peak broadening varies inversely with crystallite size [25]. According to the phase pattern obtained in Figure 4, the grain size significantly increased along the Z-direction as energy density increase to 50 J/mm^3^, before becoming stable at a specific value, which agrees with microstructure observations in Figure 2. The XRD phase pattern in Figure 5 illustrates the peak broadening comparison along the XY plane, where a slight difference of the crystal size is observed between the samples fabricated at different SLM parameters. This corresponds to the SEM observations in Figure 3. By comparing the peak broadening of the same sample along the Z-direction and the XY plane, a significant difference can be seen in peak broadening and intensity. This difference might result from the change in the crystal shape and size, and microstrain [25,26]. The microstructure is inhomogeneous along different orientations. For more accurate values, an full width half maximum (FWHM) analysis was performed according to the phase pattern in Table 3. The results showed a broadened peak of Al and Si in the AS8 sample at the lower energy density, with FWHM values of 0.2111 and 0.5935 degrees respectively. This confirms the finer microstructure observed at the lower rates of energy densities in Figure 2. The significant difference of Al and Si peak broadening in the AS8 sample along the XY plane and Z-direction also confirms the microstructure inhomogeneity at the low energy of 27 J/mm^3^. There is no significant difference between the FWHM values detected along the top and side orientations of the AS1 sample produced at a higher energy density of 65J/mm^3^. A homogeneous equiaxed grain structure is present along the XY plane and Z-direction of the AS1 sample, which indicates an improvement in the microstructure homogeneity at the higher energy densities. A Rietveld analysis was performed to detect the relative weight percentage of Al and Si according to the XRD phase pattern measured along the top and side surfaces of the AlSi10Mg samples. The results listed in Table 4 indicate that Si becomes more soluble inside the Al matrix along the XY plane as energy density gets higher. The percentage of Si solubility inside the Al matrix is higher along the Z-direction compared to that in the XY plane for AS1 and AS3 samples after an energy density of 27 J/mm^3^ and 50 J/mm^3^ is respectively applied. In addition, the highest percentage of Si precipitates is obtained at the AS8 sample produced at the higher energy density of 65 J/mm^3^. These results validate the thickness increase of the Si network at higher energy densities in Figure 2 and Figure 3. It is worthwhile to note that Rietveld analysis was used to get the indication of the Si solubility change inside the Al matrix along the SLM process parameters applied, where the small amount of Mg_2_Si existed can be neglected in this case [18].

**Figure 6:**
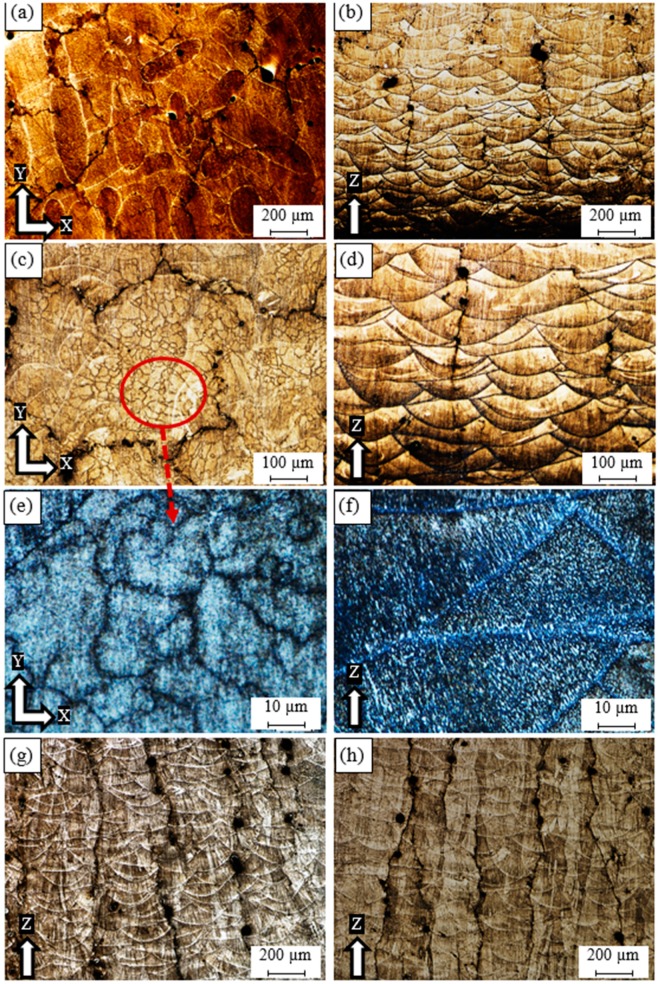
Microstructure of the as-built Al6061 samples processed under different SLM process parameters; (**a**,**c**,**e**) 6A along the Z-direction; (**b**,**d**,**f**) 6A along the XY plane; (**g**) 14A, and (**h**) 15A.

The microstructure of Al6061 samples shows hot crack formation in both the XY plane and Z-direction, as displayed in Figure 6. These cracks form as a result of solidification shrinkage and thermal contraction, or liquation cracking inside the partially melted zone [3,27]. For the 6A sample, hot cracks are observed along the XY plane within a size of 200–300 µm, and these cracks are connected in a closed loop, as illustrated in Figure 6a,c. The micro-cracks form into an elongated shape within an average size of 200 µm along the Z-direction and propagate through the middle zone of some solidified melt pool shape as shown in Figure 6b,d. A pore of 10–20 µm is also noticed amongst these cracks. The micro-crack formation is caused by high CTE of the Al6061, which, in turn, resulted in significant shrinkage due to the rapid melting and solidification rates of the SLM process [13]. A fine grain structure persists along both XY-plane and Z-direction, as shown in Figure 6e,f. Coarse grains are present around the melt pool profile, which substantiates the thermal gradient inside each melt pool during the solidification process. It is worthwhile to note that no large keyhole pores are observed inside the 6A sample microstructure fabricated with an energy density of 52.6 J/mm^3^. The evolution of crack formation behaves differently along the Z-direction, corresponding to the applied energy density and SLM process parameters, as shown in Figure 6b,g,h. Observations indicate an increase of the crack size and distribution density under higher levels of energy densities as illustrated in Figure 6g. Large hydrogen spherical pores were seen forming along the longitudinal micro-cracks as energy density increased. By comparing the microstructure in Figure 6b,h, it can be concluded that a higher laser power and lower scan speed significantly increases the length of the cracks and their distribution density due to the imbalance between the higher melting and lower solidification rates.

**Figure 7:**
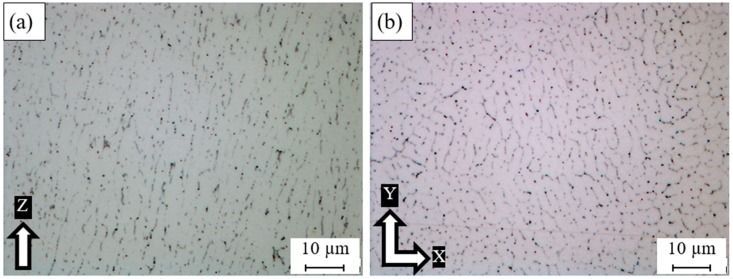
Microstructure grains of the as-built Al6061 sample at a higher magnification: (**a**) along the Z-direction; (**b**) along the XY plane.

The as-built Al6061 microstructure in Figure 7 shows the precipitation of nano-size Si particles around the Al matrix grains, which confirms the PAS formation mechanism where the Si particles solidified around the Al matrix [21]. However, the same fibrous Si network is not present in the AlSi10Mg due to Si content in the Al6061 alloy being insufficient to develop this fibrous network. A fine microstructure with an elongated grain form is observed along the Z-direction with a size of 3–5 µm as shown in Figure 7a. Along the XY plane, an equiaxed grain structure is present, with an average grain size of (2–4 µm), Figure 7b. The difference in the grain structure between these orientations reveals microstructure inhomogeneity, which could result in anisotropic structure properties.

**Figure 8:**
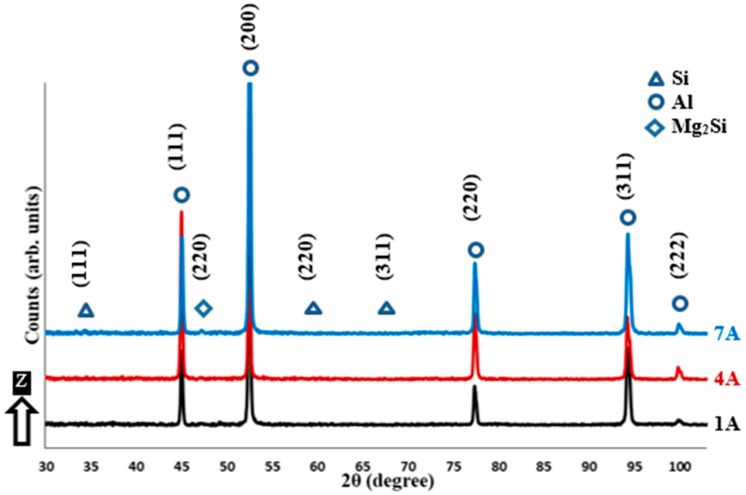
XRD phase pattern measured on the side surface (along the Z-direction) of different as-built Al6061 samples.

**Figure 9:**
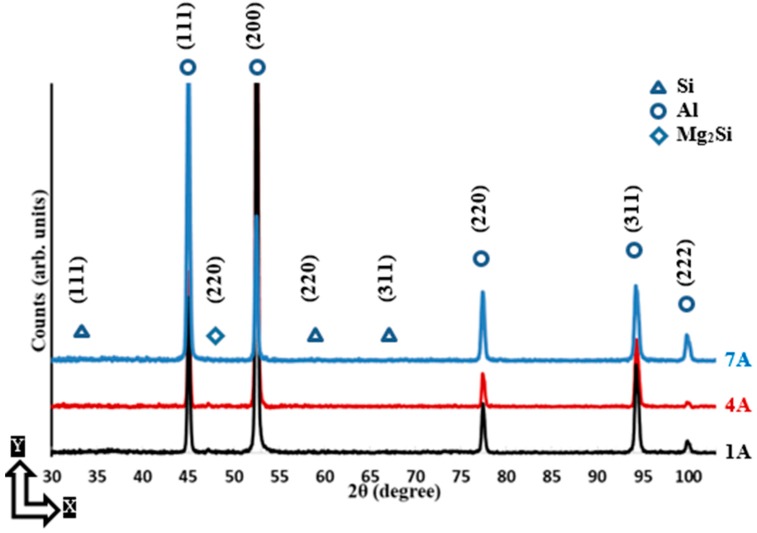
XRD phase pattern measured on the top surface (along the XY plane) of different as-built AlSi10Mg samples.

The XRD phase pattern in Figure 8 shows Al and Si peak up on the top surface of the as-built Al6061 samples in the XY plane. Figure 9 illustrates the phase pattern up on the side surface along the Z-direction. The Al peak is identified according to the JCPDS pattern of 01-089-2837. According to the JCPDS patterns of 01-089-5012, a Si peak was hardly distinguishable due to the precipitation of the nano-size Si particles inside the as-built microstructure, as displayed in Figure 7. A low-intensity peak of Mg_2_Si is also detected according to the JCPDS pattern of 00-001-1192, as indicated in Figure 8 and Figure 9. The change of Al peak broadening along the XY plane and Z-direction indicates Al crystal size change according to the specified SLM process parameters. This change is closely investigated, according to FWHM analysis listed in Table 5. A wider Al peak is obtained at a low energy density of 50 J/mm^3^, which confirms the growth of the grain size as energy density increases. According to Scherrer’s equation, the sharper peak in the XRD phase pattern indicates a larger crystal size [25]. The FWHM shows a lower value of 0.1874 degrees in the 1A sample produced at an energy density of 123.3 J/mm^3^, revealing a coarser grain structure at higher energy densities. There was no significant difference between the FWHM values of the top and side surfaces. Al6061 microstructure is more homogeneous along the applied range of the selected parameters as compared to the considerable microstructure inhomogeneity inside AlSi10Mg samples. It is worthwhile to note that the Al6061 alloy could be processed at higher energy density values than the AlSi10Mg alloy due to the higher reflectivity of Al6061, which decreases the percentage of absorbed energy. However, SLM process parameters need to be optimized to reduce the formation of micro-cracks and the spherical hydrogen pores.

### 3.2. Mechanical Properties

The effect of SLM process parameters on the mechanical properties of the as-built AlSi10Mg and Al6061 parts is investigated according to the regression models developed from experimental results. In the following section, DOE analysis will illustrate microhardness and tensile behavior according to the selected SLM process parameters.

#### 3.2.1. Microhardness

**Figure 10:**
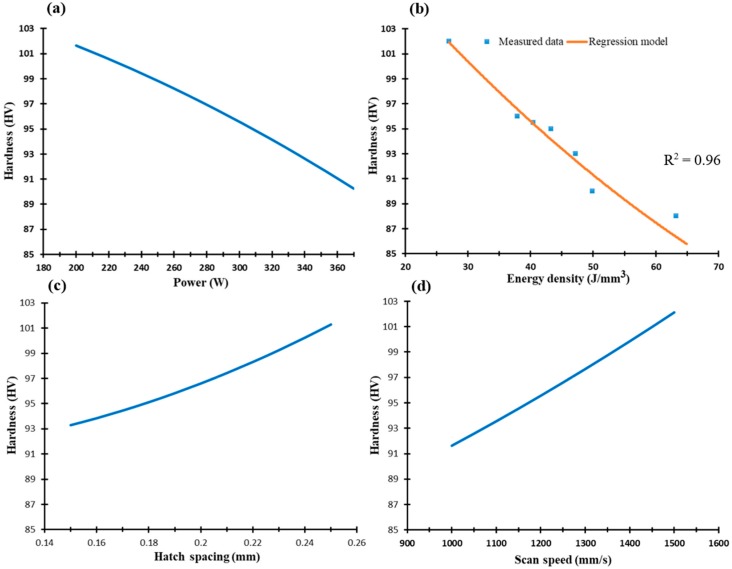
Microhardness of the as-built AlSi10Mg samples along the Z-direction vs. (**a**) Laser power (W); (**b**) Energy density (J/mm^3^); (**c**) Hatch spacing (mm), and (**d**) Scan speed (mm/s).

Figure 10 displays the microhardness of the as-built AlSi10Mg parts along the Z-direction within the range of SLM process parameters. Microhardness ranges between 86 and 103 HV; the maximum value is obtained at 27 J/mm^3^, due to smaller grain size, as presented in Figure 2b. However, a significant number of keyhole pores are observed at this energy density of the AS8 sample, which underscores the need for SLM process optimization. The results show that microhardness values linearly decrease as laser power and energy density grow, as illustrated in Figure 2a,b. A higher hatch spacing and scan speed improve sample microhardness in Figure 10c,d. Low values of sample microhardness at low scan speeds result from high solidification rates and low hatch spacing due to decreasing overlap between the scanned passes [3,24]. The microhardness profile of AlSi10Mg samples shows a good agreement with microstructure observations and the crystal size change of SLM process parameters. 

**Figure 11:**
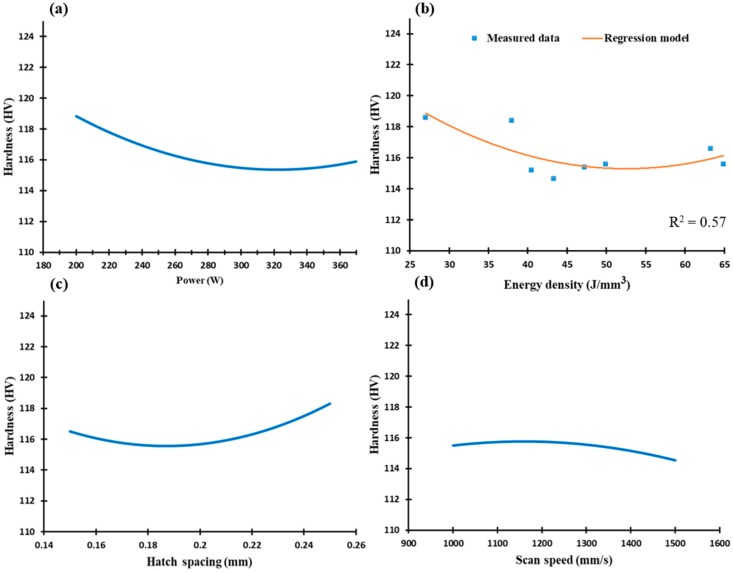
Microhardness of the as-built AlSi10Mg samples along the XY plane vs. (**a**) Laser power (W); (**b**) Energy density (J/mm^3^); (**c**) Hatch spacing (mm), and (**d**) Scan speed (mm/s).

As illustrated in Figure 11, microhardness along the XY plane is relatively higher than in the Z-direction, demonstrating the inhomogeneity of the as-built microstructure. The microhardness is 115 to 118 HV along the range of the SLM parameters, which confirms better homogeneity along the XY direction, Figure 3. This trend agrees with studies in the literature [18,20,22]. The reduction in laser power and greater hatch spacing improves microhardness along the XY plane, as shown in Figure 11a,c. Although the low laser power rates show higher microhardness values, control of SLM process parameters should aim to produce denser parts by reducing porosity. According to Figure 10 and Figure 11, microhardness values correspond to the DOE analysis regression model along both the XY plane and Z-direction. 

**Figure 12:**
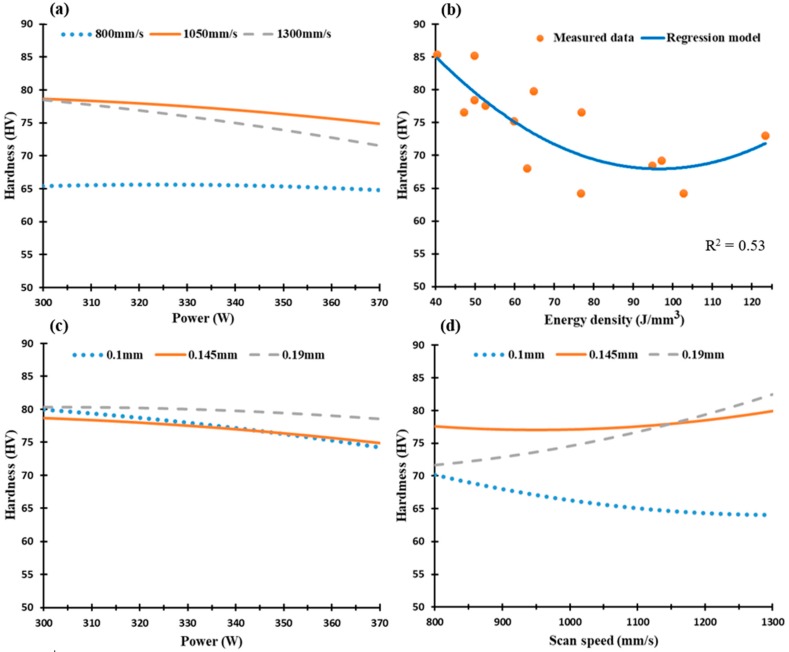
Microhardness of the as-built Al6061 samples along the Z-direction vs. (**a**) Laser power (W); (**b**) Energy density (J/mm^3^), (**c**) Hatch Spacing (mm), and (**d**) Scan speed (mm/s).

**Figure 13:**
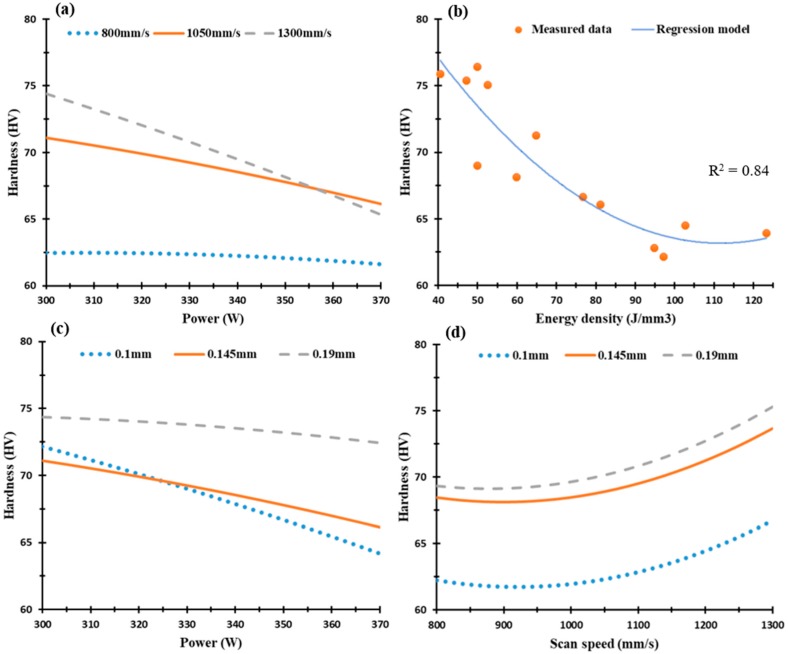
Microhardness of the as-built Al6061 samples along the XY plane vs. (**a**) Laser power (W); (**b**) Energy density (J/mm^3^); (**c**) Hatch Spacing (mm), and (**d**) Scan speed (mm/s).

Figure 12 and Figure 13 display the microhardness profile of selected SLM process parameters of the Al6061 parts along the Z-direction and XY plane respectively. The map in Figure 12 shows a gradual decrease of microhardness values along the Z-direction from 85 HV to 72 HV at an energy density range of 40.5 J/mm^3^ to 97.2 J/mm^3^. A slight increase was observed at higher energy densities, e.g., up to 123 J/mm^3^, as illustrated in Figure 12b. At a microhardness of 78 HV, a relation is observed between the low laser power of 300 W and scan speeds of 1050 mm/s and 1300 mm/s, Figure 12a. Scan speeds higher than 800 mm/s show a significant increase in microhardness due to the associated higher rate of solidification as illustrated in Figure 12a. Results indicate that a finer microstructure can be obtained at these higher scan speeds. Another interaction between scan speed and hatch spacing occurs at a scan speed of 1050 mm/s and hatch spacing values of 0.145 mm and 0.19 mm at a microhardness value of 77 HV, as shown in Figure 12d. The average microhardness measure has a high scattering pattern around the regression model due to the effect of the micro-cracks formed inside the parts, and thus, results from the microstructure inhomogeneity along the Z-direction [18]. 

Figure 13 shows microhardness of the Al6061 samples along the XY plane that varies significantly between 62 HV to 77 HV according to the SLM process parameters. This could be related to the change in micro-crack size, as illustrated in Figure 6. Figure 13a,b shows microhardness decrease along with laser power and energy density increase due to increasing the solidification rate, and thus results in a more coarser grain structure [20]. Figure 13c,d illustrates microhardness increase along with increasing scan speed and reducing laser power, and thus indicates that energy density is the leading parameter affects this property. In contrast with AlSi10Mg samples, hatch spacing significantly affects the microhardness of Al6061. Microhardness gradually decreases with the increase of energy density due to the gradient in microstructure characteristics. This is in agreement with the trend reported in studies in the literature [12,20].

Due to greater Si content, the microhardness of AlSi10Mg samples was significantly higher than that of Al6061 samples. The as-built AlSi10Mg samples have a higher microhardness than the same alloy cast material, which is limited to 75 HV [28]. The particle size distribution of the powder and its shape also might affect the microhardness of the as-built parts. This was demonstrated by comparing the microhardness values in this study with those reported by Maamoun et al. at different powder characteristics [18]. 

#### 3.2.2. Tensile Properties

**Figure 14:**
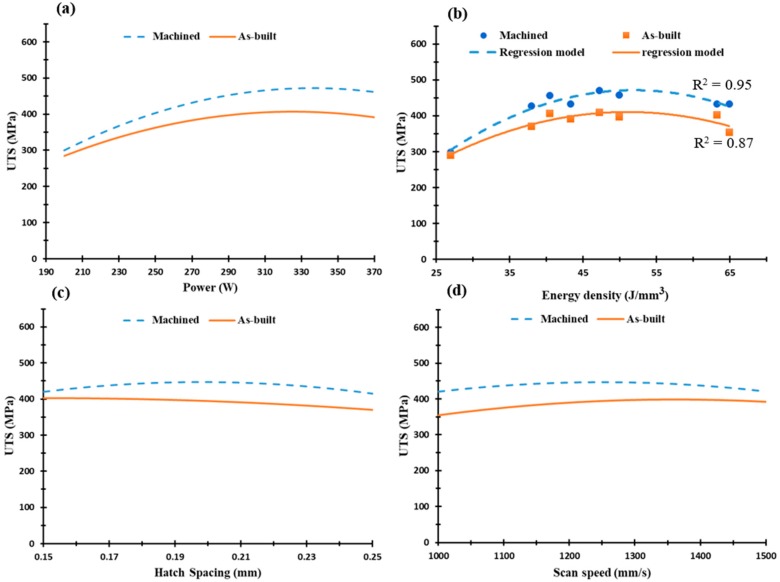
Ultimate tensile strength of the as-built AlSi10Mg samples along the building direction vs. (**a**) Laser power (W); (**b**) Energy density (J/mm^3^), (**c**) Hatch Spacing (mm), and (**d**) Scan speed (mm/s).

The ultimate tensile strength (UTS) of the AlSi10Mg was measured to generate the regression model plots for both as-built and machined tensile samples, as presented in Figure 14. The as-built and machined samples possessed the same tensile profile as the samples produced under SLM process parameters. However, the machined samples had higher UTS values of up to 450 MPa compared to those of the as-built samples (400 MPa). This 20 MPa to 50 MPa difference in UTS values indicates the effect of surface roughness on mechanical properties. However, UTS values of the as-built parts could demonstrate the impact of SLM parameters on tensile properties, taking into consideration the surface roughness of each sample. Figure 14 also shows a good agreement between the experimental measurements and the regression model generated from the DOE analysis, as illustrated in Figure 14b. Also, laser power has a more significant effect on UTS sample properties than changes in hatch spacing and scan speed, Figure 14a,c,d. The optimum UTS value is obtained in the AS3 sample at an energy density of 50 J/mm^3^. This agrees with the microstructure observation, which showed minimum defects of the as-built AlSi10Mg sample at these parameters. It is worthwhile to note that better surface roughness of the as-built AlSi10Mg was obtained at higher energy density that 50 J/mm^3^ [16]. However, the current results indicate that applying an energy density of 50 J/mm^3^ is the optimum condition for processing AlSi10Mg alloy using a scan speed of 1300 mm/s, 370 W laser power, and 0.19 mm hatch spacing. This could result in better quality of the as-built AlSi10Mg parts according to the mutual connection between surface roughness, microstructure, and mechanical properties. 

**Figure 15:**
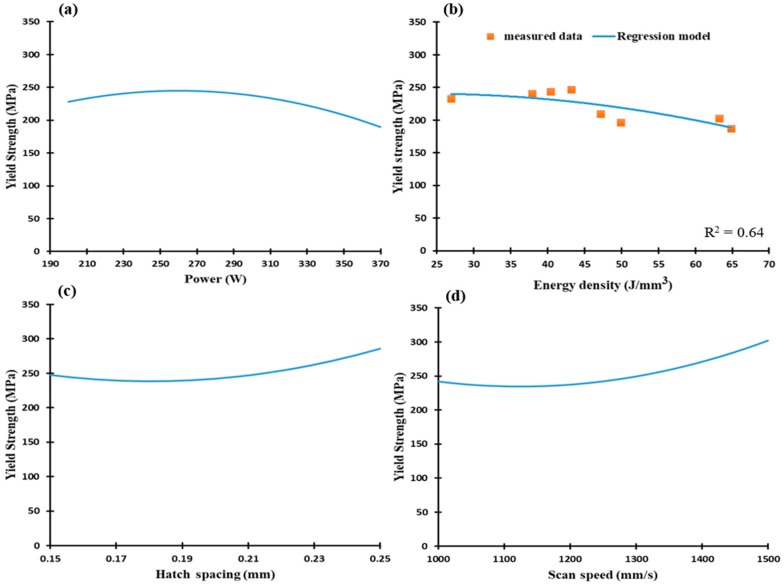
Yield strength of the as-built AlSi10Mg samples vs. (**a**) Laser power (W); (**b**) Energy density (J/mm^3^); (**c**) Hatch Spacing (mm), and (**d**) Scan speed (mm/s).

Figure 15 illustrates yield strength versus the scan speed, laser power, hatch spacing, and the energy density for the as-built AlSi10Mg samples. Results indicate a decrease of yield strength within a range of 240 MPa to190 MPa at increasing energy densities as presented in Figure 15b. A slight difference of 30–50 MPa in yield strength was observed at the range of SLM process parameters, Figure 15a–d. This indicates that a change in SLM process parameters has a greater impact on UTS values than the yield strength. From the results illustrated in Figure 14 and Figure 15, UTS and yield strength trends of the s-built AlSi10Mg parts significantly reflect the microstructure observations in Section 3.1. An increase of energy density creates a coarser microstructure with lower hardness and tensile values. This trend is in agreement with that reported by Ding et al. [29]. Moreover, some results obtained in the current study showed superior values of mechanical properties than those reported in the literature [29,30,31].

**Figure 16:**
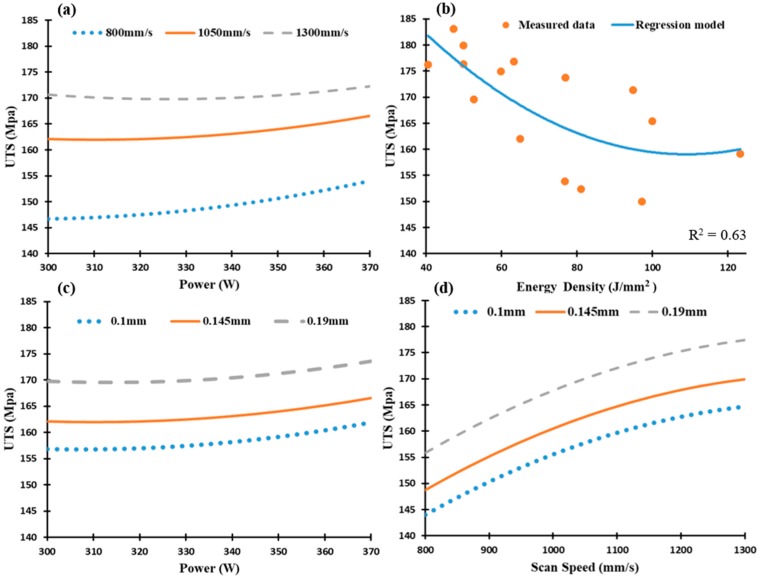
Ultimate tensile strength of the as-built Al6061 samples along the building direction vs. (**a**) Laser power (W); (**b**) Energy density (J/mm^3^), (**c**) Hatch Spacing (mm), and (**d**) Scan speed (mm/s).

As illustrated in Figure 16a–d, the UTS values of the as-built Al6061 samples were investigated at a range of 150 MPa to 184 MPa. The results indicate a significant reduction in UTS of the Al6061 samples compared to that of AlSi10Mg. This could result from the lower percentage of Si content inside the Al6061 alloy and micro-cracks inside its as-built samples. As the energy density increases, UTS values gradually decrease. Figure 16b shows that a maximum UTS of 184 MPa was obtained in the 18A sample using the higher scan speed (1300 mm/s), hatch spacing (0.19 mm), and energy density of 47.2 J/mm^3^. A significant decrease in the UTS values was observed at the lower scan speed of 800 mm/s and smaller hatch spacing of 0.1 mm, Figure 16a,c,d. This decrease in the UTS values might result from the microstructure defects at low rates of scan speed and hatch spacing, such as keyhole pores or areas of unmelted powder. 

**Figure 17:**
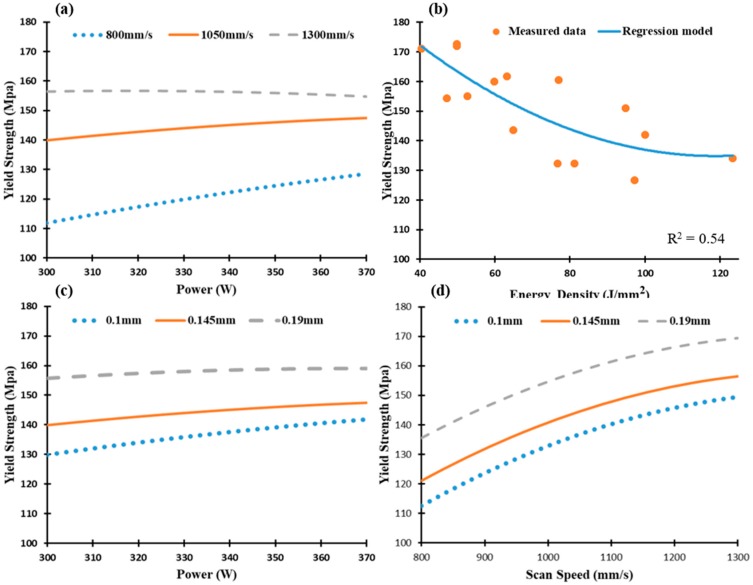
Yield strength of the as-built Al6061 samples vs. (**a**) Laser power (W); (**b**) Energy density (J/mm^3^); (**c**) Hatch Spacing (mm), and (**d**) Scan speed (mm/s).

Yield strength of the Al6061 samples is presented in Figure 17, where a similar trend as in UTS is present. The yield strength values along the SLM parameters vary from 125 MPa to 172 MPa, Figure 17a–d). The maximum yield strength of 172 MPa was detected in the 8A and 18A samples also produced at the higher scan speeds, hatch spacing and energy density range of 40.5–47.2 J/mm^3^. The trend obtained for the mechanical properties behavior shows a good agreement with that reported in some previous studies [13,14,32], in addition to detecting the values of SLM parameters that results in a higher values than reported in these studies. It is worthwhile to note that the UTS and yield strength values of the as-built Al6061 samples hardly differ, which indicates the lower ductility of these parts compared to the as-built AlSi10Mg samples.

**Figure 18:**
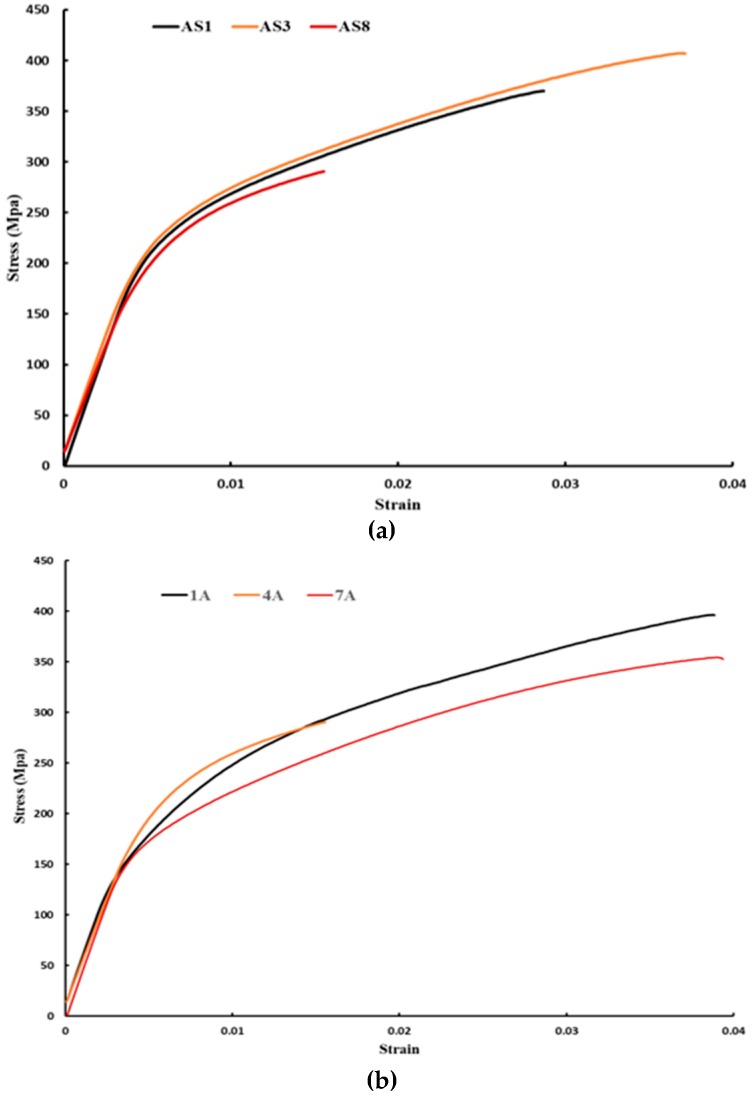
The stress strain diagram for the as-built samples: (**a**) AlSi10Mg; and (**b**) Al6061 samples.

Figure 18 shows the stress-strain curve of the as-built samples for both AlSi10Mg and Al6061 alloys. Figure 18a illustrates the stress-strain behavior of the AS1, AS3, and AS8 AlSi10Mg samples. The maximum UTS and highest ductility was observed in the AS3 sample produced at an energy density of 50 J/mm^3^. Microstructure observations confirm that the optimum SLM process parameters of the AlSi10Mg alloy are present in the AS3 sample. The AS1 sample was affected by hydrogen pores and a coarse microstructure that forms at a higher 65 J/mm^3^ energy density, resulting in lower stress value. Keyhole pores and lack of fusion negatively affected the quality of the AS8 sample produced at a low energy density of 27 J/mm^3^, which resulted in the lowest material strength along with higher brittleness. The strain curves of the 1A, 4A, and 7A Al6061 samples are presented in Figure 18b. Energy density change had no significant effect on the UTS value, whereas laser power proved to be the most influential. The 4A sample produced at a low laser power level of 300 W, exhibited minimum UTS values.

Table 6 summarizes the mechanical property values of the AlSi10Mg and Al6061 samples in the current study, compared to the literature. According to values listed in Table 6, the following insights can be drawn:

1. Mechanical properties and Al matrix grain size are illustrated for the as-built AlSi10Mg_200C samples in the current study. Although the lower rate of energy density created a fine microstructure, mechanical properties were inferior due to the internal defects inside the areas caused by lack of fusion. 

2. The microhardness reported in a previous study by the authors [18], using the same preheating technique, shows higher values than those reported in this study. This indicates the effect of powder morphology and its particle size distribution. It can be concluded that a wide range of particle size distribution with a spherical shape resulted in high microhardness values.

3. The mechanical properties of the AlSi10Mg_200C samples have relatively lower values than those of samples produced by build plate preheating [30,33,34,35,36]. However, residual stresses are significantly lower due to the preheating technique [4,18]. 

4. Superior mechanical properties of the AlSi10Mg_200C samples are detected compared to parts produced with a conventional or the high-pressure die cast (HPDC) material of the same alloy [28,37]. 

5. As-built Al6061_200C parts had better mechanical properties than Al6061_500C. However, no cracks were observed inside the Al6061_500C as reported by Uddin et al. [14], but the mechanical properties of the part were significantly decreased. 

6. The mechanical properties of the Al6061_200C samples show comparable values to the T6, and T4 treated Al6061 wrought material [38]. 

## 4. Summary and Conclusions

The current study focused on the influence of SLM process parameters on the microstructure and mechanical properties of the as-built AlSi10Mg and Al6061 parts. The mechanical behavior of these parts along the range of selected SLM parameters was investigated using DOE regression models. The main results are summarized as follows:

1. The microstructure of the AlSi10Mg parts changes significantly according to the applied energy density. After solidification, the size of the melt pool profile increases together with energy density. An energy density range of 50–60 J/mm^3^ was found to be the optimal range of the energy density due to it minimizing keyholes and larger hydrogen spherical pores. 

2. The grain size of the Al matrix inside the as-built AlSi10Mg samples grows along with energy density. The microstructure homogeneity is also improved by the development of an equiaxed grain structure at 65 J/mm^3^ along the Z-direction and XY plane. However, this can adversely affect the relative density due to the formation of large hydrogen pores. 

3. Micro-cracks form inside the microstructure of the as-built Al6061 samples. Size and distribution of these cracks vary according to SLM process parameters. The smallest micro-cracks are obtained at an energy density of 52.6 J/mm^3^ and a scan speed of 1000 mm/s.

4. The microstructure of Al6061 parts did not show the same fibrous Si network that formed inside the AlSi10Mg microstructure due to lower Si content in the Al6061 alloy. The microstructure of Al6061 parts followed the PAS mechanism, and nano-size Si particles precipitated along the grain boundary of the AL matrix. 

5. Microhardness of AlSi10Mg and Al6061 parts corresponds with microstructure observations along the Z-direction and in the XY plane. However, Al6061 microhardness is affected by already present micro-cracks.

6. UTS and yield strength of the as-built AlSi10Mg and the Al6061 samples are investigated through regression models. 

7. The effect of surface texture on UTS of the AlSi10Mg parts was investigated by comparing the results from the as-built and machined tensile samples. 

8. The mechanical properties of the studied Al alloys showed different values according to the SLM process parameters, build plate temperature, powder characteristics, and the technique used in Table 6.

The current work, together with presented by Maamoun et al. [16], forms a comprehensive study of the SLM process parameters effect on the quality of Al alloy parts. The results of this study could help customize the properties of the parts according to design and function requirements. This work may also offer a means to reduce the post-processing treatment required for part characteristics in some applications.

## Figures and Tables

**Table 1:** The SLM process parameters applied for producing the AlSi10Mg_200C samples.

Sample	P (W)	V_s_ (mm/s)	D_h_ (mm)	E_d_ (J/mm^3^)
AS1	370	1000	0.19	65
AS2	370	1300	0.15	63.2
AS3	370	1300	0.19	50
AS4	350	1300	0.19	47.2
AS5	370	1500	0.19	43.3
AS6	300	1300	0.19	40.5
AS7	370	1300	0.25	38
AS8	200	1300	0.19	27

**Table 2:** The SLM process parameters used for building the Al6061_200C samples.

Sample	P (W)	V_s_ (mm/s)	D_h_ (mm)	E_d_ (J/mm^3^)	Sample	P (W)	V_s_ (mm/s)	D_h_ (mm)	E_d_ (J/mm^3^)
1A	370	1000	0.1	123.3	11A	370	800	0.15	102.8
2A	300	1000	0.1	100	12A	350	800	0.15	97.2
3A	370	1300	0.1	95	13A	370	800	0.19	81.1
4A	300	1300	0.1	76.9	14A	350	800	0.19	76.8
5A	370	1000	0.19	65	15A	370	1300	0.15	63.2
6A	300	1000	0.19	52.6	16A	350	1300	0.15	59.8
7A	370	1300	0.19	50	17A	370	1300	0.19	50
8A	300	1300	0.19	40.5	18A	350	1300	0.19	47.2

**Table 3:** The average FWHM of Al and Si peaks according to the XRD phase pattern of the as-built AlSi10Mg samples.

Material	Peak	Position	AS1	AS3	AS8
AlSi10Mg	Al (200) FWHM (°)	Top (XY)	0.2111	0.2332	0.2294
		Side (Z)	0.2105	0.2304	0.2269
	Si (220) FWHM (°)	Top (XY)	0.5935	0.7281	0.7137
		Side (Z)	0.5217	0.5531	0.5420

**Table 4:** Rietveld analysis throughout the top and side surfaces of the as-built AlSi10Mg samples.

Material	Element	Top Surface (XY plane)			Side Surface (Z-direction)		
		AS1	AS3	AS8	AS1	AS3	AS8
AlSi10Mg	Al wt.%	91.11	91.98	90.81	93.49	93.57	90.75
	Si wt.%	8.89	8.02	9.19	6.51	6.43	9.25

**Table 5:** The average FWHM of Al (200) peak of the as-built Al6061 samples.

Material	Peak	Position	1A	4A	7A
Al6061	Al (200) FWHM (°)	Top (XY)	0.1874	0.2086	0.2045
		Side (Z)	0.1838	0.2042	0.2029

**Table 6:** A summary of mechanical properties microstructure grain size of the AlSi10Mg and Al6061 parts processed though SLM and the conventional techniques under different conditions.

Material	SLM Process Parameters			Energy Density (J/mm^3^)	Treatment	UTS (MPa)	Yield Strength (MPa)	Average Hardness (HV)	Al Matrix Grain Size (µm)
	P (W)	Vs (mm/s)	Dh (mm)						
AlSi10Mg_200C (Current Study)	370	1000	0.19	65	As-built	354.6	186.4	Z 102	3–4
								XY 118	0.3–2
	370	1300	0.19	50	As-built	396.5	196	Z 90	0.5–3
								XY 115	0.3–2
	200	1300	0.19	27	As-built	290.6	232.3	Z 84.5	0.2–2
								XY 116	0.15–1
AlSi10Mg_200C [18]	370	1300	0.19	50	As-built			Z 120	0.5–1
								XY 130	
					T6			Z 115	1–5
								XY 116	
AlSi10Mg [36]	250	500	0.5		As-built	350	250	145	
					T6	285	340	116	
AlSi10Mg [33]					As-built	460 ± 20	270 ± 10	119 ± 5	
AlSi10Mg_200C [34]					As-built	390	210		
AlSi10Mg [30]	200	1400	0.105		As-built	391 ± 6		127	
AlSi10Mg [35]	350	1650	0.13	54.4	As-built	412 ± 2	242 ± 5	139	
AlSi10Mg [37]					HPDC	300–350	160–185	95–105	
					HPDC-T6	330–365	285–330	130–133	
A360 [28]					Casting	317	165	75	
Al6061_200C (Current Study)	370	1000	0.1	123.3	As-built	396.5	196	Z 67	4–6
								XY 71	4–5
	300	1300	0.1	76.9	As-built	290	232.3	Z 81	4–5
								XY 77	3–4
	370	1300	0.19	50	As-built	392	246.7	Z 67	3–5
								XY 84	2–4
Al6061 [14,32]	400	1400	0.14	20.41	As-built			90 ± 6	
Al6061_500C [14]					As-built	133	66	54 ± 2.5	
					T6	308	282	119 ± 6	
AA6061-wrought [38]					O	125	55	30	
					T4	240	145	65	
					T6	310	276	95	
